# Household Management of Childhood Diarrhoea: A Population-based Study in Nicaragua

**Published:** 2014-03

**Authors:** Sylvia Becker-Dreps, Luis Enrique Zambrana, Daniel Reyes, Samuel Vilchez, David J. Weber

**Affiliations:** ^1^Department of Family Medicine, University of North Carolina School of Medicine, Chapel Hill, North Carolina, USA; ^2^Center for Epidemiology and Health (CIDS), León, Nicaragua; ^3^Department of Microbiology and Parasitology, National Autonomous University of Nicaragua, León, Nicaragua; ^4^Division of Infectious Diseases, University of North Carolina School of Medicine, Chapel Hill, North Carolina, USA

**Keywords:** Child, Diarrhoea, Household management, Nicaragua

## Abstract

Diarrhoea remains an important cause of mortality and morbidity among children in Nicaragua. As the majority of diarrhoeal cases are treated at home and appropriate household management can lessen severity of diarrhoea, the objective of this study was to examine household management of childhood diarrhoea. A simple random sample of households was selected from the Health and Demographic Surveillance Site-León. Parents or caretakers of children below five years of age, who developed diarrhoea (n=232), were surveyed about household diarrhoea management practices in 2011. Fifty-seven percent of children received oral rehydration therapy (ORT) in the home prior to visiting any health facility. We encountered certain practices in contradiction with WHO recommendations for the management of diarrhoea in communities: 41% of children were offered protein-rich foods less frequently during diarrhoeal episodes, 20% of children were nursed less frequently or not at all during diarrhoeal episodes, and zinc supplementation was recommended at only 39% of visits with healthcare providers. Our findings provide insights for efforts to improve the household management of childhood diarrhoea in Nicaragua.

## INTRODUCTION

Diarrhoeal diseases are the second leading cause of childhood mortality worldwide (1) and can contribute to malnutrition and developmental delays (2,3). The great majority of diarrhoeal episodes are treated at home (4). Optimizing the management of diarrhoea in the household setting may lessen the severity and sequelae of diarrhoeal episodes (5).

To this end, the World Health Organization (WHO) has provided recommendations on the management of childhood diarrhoea by families and in communities (6). These recommendations include early initiation of oral rehydration therapy (ORT); continuation of breastfeeding; continuation of high-energy, micronutrient-rich diet; zinc supplementation; and the need to seek medical care as appropriate. While the recommendations are clear, little is known about adherence to these recommended practices to inform health education efforts. Data on the use of ORT are provided for many countries by Demographic and Health Surveys (DHS) (7) but less is known about dietary restrictions and appropriate use of zinc and antibiotics for children with diarrhoea.

In Nicaragua, a low/middle-income country in Central America, diarrhoea remains among the most common causes of child mortality and morbidity (8-10). Prior studies on the household management of diarrhoea in the region are limited. DHS from Nicaragua show that more than one-half (54%) of caretakers had provided ORT to their children with diarrhoea; however, 52% had provided the child with less food, and 25% had provided the child with less fluids during the diarrhoeal episode. A study on the household management of diarrhoea in the Dominican Republic reported high rates of dietary restrictions (11); another study from peri-urban Mexico found that self-medication with antibiotics for diarrhoea management was common (12).

As the majority of diarrhoea cases are treated at home and appropriate household management of diarrhoea can lessen the severity of diarrhoeal episodes, the objective of this study was to examine the household management of childhood diarrhoea in Nicaragua. To examine the diarrhoea management in this setting, we surveyed parents or caretakers of a population-based sample of children below five years of age.

## MATERIALS AND METHODS

### Setting

The study was conducted in León, Nicaragua's second largest city, having an estimated population of 193,123 in 2011. During the time the study was conducted, the population of León received a municipal water supply (chlorinated groundwater).

Primary healthcare in León is provided at (i) government-administered (public) primary-care centres and health posts, which provide services free of charge; (ii) fee-for-service private physicians’ offices; and (iii) C*línicas previsionales,* a tier of private clinics providing care for insured government workers and others with health insurance.

### Sample selection, design, and data collection

The Health and Demographic Surveillance Site-León (HDSS-León) maintains demographic surveillance for 10,994 households in the municipality of León (13). A simple random sample of 531 households was selected from HDSS-León as part of a larger study on childhood diarrhoea incidence (14). Of the 864 children below five years of age, who resided in selected households over the course of the one-year study, parents of 826 children consented for participation in the study. The study followed an ‘open cohort’ design; new children and newborns moving into selected households were enrolled while children who passed their fifth birthday or moved out of selected households no longer contributed to data.

Field workers visited household**s** to collect baseline characteristics and weight of children and returned every two weeks over a one-year study period (25 January 2010 to 24 January 2011) to record information on diarrhoeal episodes (14). During the final two months of the year-long study, 618 children remained under surveillance (neither had passed their fifth birthday nor moved out of a selected household). Parents or caretakers of children under surveillance (N=618), who had developed diarrhoea (N=232), were administered an additional questionnaire on household diarrhoea management. All field workers were female and had been trained on questionnaire administration. Quality control of field data collection was performed by the field supervisor with systematic and random evaluations. While some children had developed multiple diarrhoeal episodes, parents or caretakers were administered the questionnaire only once per child. The questionnaire included questions on ORT initiation, antibiotic usage (directly recording information from medication bottles whenever available), breastfeeding frequency, recommendations from healthcare providers, and, finally, a list of foods that were offered ‘more frequently’, ‘the same as usual’, ‘less frequently’, or ‘not at all’ during the diarrhoeal episode.

In this study, diarrhoea was defined as an increase in stool frequency to at least three stools per 24-hour period or a substantial change in stool consistency to loose, watery, or bloody, following at least three diarrhoea-free days. Socioeconomic status was assessed by a validated poverty index—Unsatisfied Basic Need Assessment (15); a score of 0 or 1 implies non-poverty, with basic needs being met while 4 suggests severe poverty.

Informed consent was obtained from a parent or legal guardian of each participant. The study was approved by the Institutional Review Boards of the University of North Carolina at Chapel Hill and the National Autonomous University of Nicaragua-León.

### Statistical analysis

We determined the frequency of each response among the study participants. Chi-square testing was used for comparing response frequency between those who received their care in government-administered vs private health facilities, to determine if providers in government-administered facilities were more likely to follow WHO recommendations. In this analysis, both fee-for-service private clinics and *clínicas previsionales* were categorized as ‘private health facilities’. Participants’ weights were converted into percentiles, using WHO weight-for-age standards (16).

## RESULTS

Sets of household diarrhoea management questionnaire were completed for all of the 232 children with diarrhoea. Characteristics of the surveyed children and their households are shown in Table 1. Among the surveyed children, 53% were female, and the mean age was 25 months. Seventeen percent of children had weight-for-age below the 10th percentile, and 28% were currently breastfed. Seventy-two percent were considered to have their basic needs met, and 82% had mothers who had completed schooling beyond the primary level.

Diarrhoeal episodes were associated with a mean of four stools per 24 hours; vomiting was reported with 29% of episodes, fever with 27% of episodes, and bloody stools with 3%. Fifty-seven percent of respondents reported providing ORT to the child in the home prior to visiting any health facility (Table 2), either as packaged oral rehydration salts (ORS, 83%) or as recommended home solution (RHS, 17%). Changes in foods offered to children during diarrhoeal episodes are shown in Table 2. Forty-one percent of parents or caretakers offered certain foods less frequently during diarrhoeal episodes. Foods offered less frequently included beans, dairy products (local cheeses and buttermilk), and eggs. Fifty-two percent of parents or caretakers offered foods more frequently during diarrhoeal episodes. Foods given more frequently during diarrhoeal episodes included broths or soups, meats, and rice or rice-water. Twenty percent of breastfed infants were offered breastmilk less frequently or were not offered breastmilk at all during the diarrhoeal episodes. Antibiotics were received by 19% of the children for diarrhoea—most commonly trimethoprim-sulphamethoxazole.

**Table 1.** Characteristics of participating children

Characteristics	Frequency (N=232)
Female sex	53% (123/232)
Age in months, mean (standard deviation)	25 (15)
Current breastfeeding	28% (65/232)
Weight-for-age >10th percentile	83% (193/232)
Indoor municipal water source in child's home	97% (225/232)
Indoor toilet in child's home	84% (195/232)
Concrete or tile floor (non-dirt floor) in child's home	80% (186/232)
Mother with education beyond the 4^th^ grade	82% (188/229)*
Basic needs met (poverty index=0 or 1)	72% (167/232)

*Three respondents declined to answer this question

Sixty-six percent of children received care for their diarrhoea from a healthcare provider. Medical advice received from healthcare providers is shown in Table 3. As per report of the parent or caretaker, 88% of healthcare providers recommended ORT for the treatment of diarrhoeal episode while 39% recommended zinc supplementation. Children who received zinc took a seven-day course, on average. The majority of healthcare providers did not recommend a change in diet during the diarrhoeal episode**s** (87%); however, among the providers who did recommend a change, only 11% recommended dietary changes that were in agreement with the WHO recommendations. We did not find a statistically significant difference in these practice patterns between private and public health facilities.

## DISCUSSION

Our population-based study of diarrhoea management practices found that almost half of the children were offered food less frequently during diarrhoeal episodes, which is in agreement with a prior study from the region (11). The foods most-commonly withheld were protein-rich foods, such as beans, dairy products, and eggs. Also, one in five breastfed children in the study was offered breastmilk less frequently or not at all during their diarrhoeal episode**s**, which has been documented elsewhere (17,18). These practices are in contradiction with WHO recommendations (6) and may contribute to a decline in nutritional status from diarrhoeal episodes. Frequency of the use of ORT among study participants (57%) was similar to that reported by Demographic Health Surveys in Nicaragua in 2001 (54%) (7). The majority (88%) of healthcare providers recommended ORT; however, less than half (39%) recommended zinc supplementation. While zinc shortens the duration of diarrhoea and prevents future episodes, uptake of this intervention has been slow (19). We did not detect a difference in practice recommendations from providers at government-run clinics compared to private clinics. Similar to the previous study in peri-urban Mexico City (12), we found a high prevalence of antibiotic use. Nineteen percent of children received an antibiotic for the treatment of diarrhoea—most commonly trimpethoprim-sulphamethoxazole. According to the Integrated Management of Childhood Illness (IMCI), antibiotics are only indicated for bloody diarrhoea, which was reported in 3% of diarrhoeal episodes. Furthermore, there is high resistance to trimethoprim-sulfamethoxazole among bacterial pathogens of diarrhoea in Nicaragua (20).

**Table 2.** Household practices in the management of childhood diarrhoea

Household diarrhoea management practice	Frequency (N=232)
Child was given ORT* in the home	57% (133/232)
Where from were ingredients for ORT obtained? (n=133)	
Pharmacy (packaged ORS)†	62% (83/133)
Government-administered public health facility(packaged ORS)	21% (28/133)
Prepared from ingredients in the home (RHS)‡	17% (22/133)
Among those currently-breastfed (n=64), child was offered breastmilk:	
More than usual	44% (28/64)
The same as usual	36% (23/64)
Less than usual	5% (3/64)
Not at all during illness	16% (10/64)
Child was given certain foods less frequently during diarrhoeal episode	41% (95/230)§
Foods given less frequently (n=95):	
Beans or *gallo pinto* (red beans and rice)	53% (50/95)
Dairy products	30% (28/95)
Eggs	28% (27/95)
Fruit	5% (5/95)
Other (meat, fried foods, vegetables, pasta, chocolate)	11% (10/95)
Child was given certain foods more frequently during diarrhoeal episode	52% (119/230)§
Foods given more frequently (n=119):	
Broth vegetable or chicken soup	55% (65/119)
Meat or fish	22% (26/119)
Rice or rice-water	18% (21/119)
Fruit or fruit drink	11% (13/119)
Vegetables	10% (12/119)
Other (tortillas, barley, gelatin)	5% (6/119)
Child received a natural home remedy (including herbal teas, guava leaf drink, cashew fruit drink, lemon-based drinks, garlic)	13% (30/232)

*Oral rehydration therapy;

†Oral rehydration salts;

‡Recommended home solution;

§ Denominator is not equal to 232 because two respondents declined to answer this question

**Table 3.** Advice received from healthcare provider on diarrhoea management

Diarrhoea management advice or practice	Frequency (N=232)
Child received care for diarrhoea from a healthcare provider	66% (154/232)
Where from did child receive care for diarrhoea (n=154)?	
Clínicas previsionales*	38% (58/154)
Fee-for-service private physician	27% (41/154)
Public primary-care centre	24% (37/154)
Public hospital emergency room	10% (15/154)
Free church-run clinic	1% (2/154)
Multiple sources	1% (1/154)
Did the healthcare provider (n=154)	
recommend ORT?	88% (136/154)
recommend zinc?	39% (60/154)
Number of the days child received zinc (n=60)	7 (SD±5, range 2-30)
Did the healthcare provider recommend a change of diet (n=154)?	13% (20/154)
Dietary change that was recommended by provider (n=19)†:	
Avoid milk	32% (6/19)
Avoid heavy or fatty foods	32% (6/19)
Avoid beans	16% (3/19)
Increase fluids	11% (2/19)
Increase fruit	5% (1/19)
Increase carbohydrates	5% (1/19)

*Private clinics providing care for insured government workers and others with health insurance;

†n=19, not 20, because one respondent declined to answer this question

### Limitations

While our study provides a population-based assessment of diarrhoea management practices, we acknowledge certain limitations. First, in our assessment of antibiotics-use, we did not determine whether the antibiotic was self-prescribed or prescribed by a medical care provider. Second, our analysis of provider practice patterns may not have been adequately powered to detect a difference in practice styles between public and private clinics. Finally, we acknowledge that there may be a ‘desirability bias’ by relying on report by the parent or caretaker.

### Conclusions

Our findings provide insights for health education efforts to improve the household management of diarrhoea in Nicaragua. Parents and caretakers should be encouraged not to withhold high-protein foods during diarrhoeal episodes, and the benefits of continued breastfeeding during diarrhoea should be emphasized. Further studies should evaluate the reasons behind low adherence to guidelines which recommend zinc supplementation and the best methods to promote the appropriate management of diarrhoea in communities.

## ACKNOWLEDGEMENTS

We would like to thank the home interviewers and the participating families for their contributions to this study. Dr. Becker-Dreps is supported by 5K01TW008401-04 from the Fogarty International Center at the US National Institutes of Health.
